# Risk factors for acromial stress fractures following primary reverse total shoulder arthroplasty: the impact of prior acromioplasty and radiographic parameters

**DOI:** 10.1016/j.jsea.2026.100016

**Published:** 2026-05-12

**Authors:** Jens Vanlommel, Pieter Vandenabeele, Dirk Petré, Saartje Defoort

**Affiliations:** a Department of Orthopedic Surgery, Sint-Jozefskliniek Izegem, Izegem, Belgium; b Department of Orthopedic Surgery, University Hospital Antwerp, Edegem, Belgium; c Department of Orthopedic Surgery, University Hospital Ghent, Ghent, Belgium

**Keywords:** Reverse total shoulder arthroplasty, Acromial stress fracture, Acromioplasty, Radiographic predictors, Post-operative complications, Bone quality, Scapular spine fracture

## Abstract

**Hypothesis/Background:**

Acromial scapular stress fractures (ASFs) are a complication after primary reverse total shoulder arthroplasty (rTSA), often causing functional impairment. The role of acromioplasty and radiographic predictors in ASF is unclear. Prior acromioplasty, acromial thickness (AT), deltoid tuberosity index (DTI), and changes in critical shoulder angle (ΔCSA) may influence ASF development.

**Methods:**

A retrospective analysis of 698 patients who underwent primary rTSA from 2017 to 2022 with a minimum 2-year follow-up was carried out. ASF were confirmed by CT and classified according to Levy et al. Demographic, surgical, and radiographic data—AT, DTI, critical shoulder angle, ΔCSA, lateralization shoulder angle, and distalization shoulder angle—were collected. Data were analyzed using Firths penalized logistic regression.

**Results:**

ASF occurred in 36 patients (5.2%), (52.8% Type I, 41.6% Type II, and 5.6% Type III). Prior acromioplasty was more frequent in ASF patients (30.6% vs. 15%, *P* = .014). ASF patients had lower AT (6.5 ± 1.6 vs. 7.7 ± 1.6 mm, *P* < .001), lower DTI (1.29 ± 0.09 vs. 1.38 ± 0.13, *P* < .001), and higher ΔCSA (4.7 ± 6.9° vs. 1.0 ± 4.9°, *P* = .001). Regression identified lower DTI (odds ratio 0.61, 95% confidence interval 0.45–0.80, *P* < .001) and higher ΔCSA (odds ratio 1.14, 95% confidence interval 1.06–1.22, *P* < .001) as independent predictors, while AT and prior acromioplasty were not significant.

**Conclusion:**

Reduced DTI and greater ΔCSA are significant independent predictors of ASF after primary rTSA. Preoperative assessment DTI and careful management of critical shoulder angle may help identify high-risk patients and mitigate the incidence of ASF.

Reverse total shoulder arthroplasty (rTSA) is a widely performed and effective procedure for various shoulder pathologies.^2^^,^^9^ Despite its overall success, rTSA is associated with several notable complications, some occurring at higher rates than in anatomical shoulder arthroplasty.^19^^,^^29^ Among these, acromial scapular stress fractures (ASFs) represent a particularly concerning complication unique to rTSA, leading to substantial functional impairment and adverse poor clinical outcomes. Even after fracture union, functional recovery often remains limited.^1^^,^^4^^,^^12^^,^^21^^,^^24^^,^^26^ Reported incidence of ASF ranges from 2.9% to 10%.^11^^,^^14^^,^^16^ Previous studies have investigated potential risk factors, including older age, female sex, osteoporosis, inflammatory arthritis, and cuff tear arthropathy (CTA).^11^^,^^15^^,^^25^ However, evidence linking prior acromioplasty to an increased risk of ASF remains inconclusive.^6^ Some authors report that history of subacromial decompression is not a risk factor for subsequent ASF.^3^^,^^8^^,^^10^ However, a recent large cohort study demonstrated findings that contradict this conclusion.^27^ Radiographic predictors such as critical shoulder angle (CSA) and post-operative lateralization shoulder angle (LSA) have been associated with ASF.^12^^,^^13^ Interpretation of radiographic predictors is further complicated by the inclusion of fractures with a traumatic origin in some studies.^12^^,^^13^ Furthermore, parameters reflecting local bone quality, such as acromial thickness (AT) and deltoid tuberosity index (DTI), have been investigated but their significance remains unclear.^23^^,^^30^^,^^31^ Given the limited evidence regarding radiographic predictors and the uncertain role of prior acromioplasty, this study aimed to evaluate demographic characteristics, history of subacromial decompression, and radiographic parameters to improve understanding and guide prevention strategies, after primary rTSA. We hypothesized that prior acromioplasty is an independent risk factor for ASF and that AT, DTI, and CSA could serve as predictors of fracture risk.

## Materials and methods

**Figure 1:**
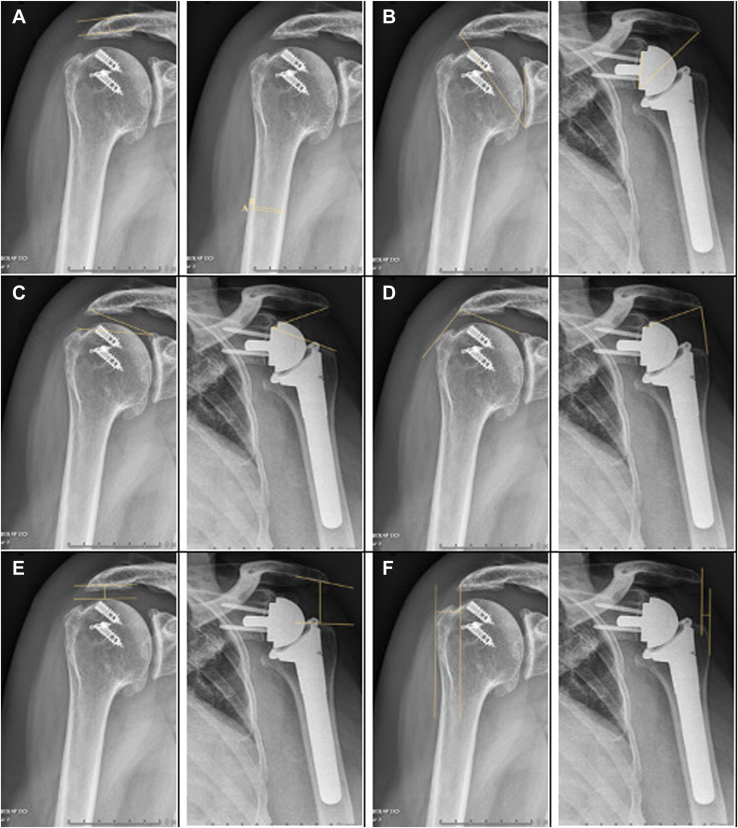
This figure is a visualization of the preoperative and post-operative radiographic parameters on a standard Anteroposterior view of the shoulder. (**A**) Acromial thickness (AT) and deltoid tuberosity index (DTI); (**B**) critical shoulder angle (CSA); (**C**) distalization shoulder angle (DSA); (**D**) lateralization shoulder angle (LSA); (**E**) acromiohumeral distance (ACHD); (**F**) distance from the lateral acromion to the most lateral acromion-to-greater tubercle distance (LAGT).

The study was approved by the local ethics committee of Sint-jozefskliniek Izegem and conducted in accordance with the Declaration of Helsinki. All patients who underwent primary rTSA between January 1, 2017, and December 31, 2022, and had a minimum of 2-year clinical and radiological follow-up were eligible for inclusion. A retrospective single-center analysis was performed. ASF were defined as the presence of post-operative acromial pain or tenderness confirmed by computed tomography. ASF were classified according to Levy et al.^14^ Type I fractures involves the midpart of the acromion corresponding to the anterior and middle deltoid origin; Type II fractures concern the entire middle of the deltoid and acromion; Type III fractures are defined as fractures extending through the posterior and middle part of the deltoid origin. All surgeries were performed by 1 of the 2 fellowship-trained senior shoulder surgeons using a standard deltopectoral approach (S.D. and D.P.). According to the surgeon's preference, the following two rTSA systems were used: the Univers Revers Total shoulder System (Arthrex, Naples, FL, USA) and the Identity Shoulder System (Zimmer Biomet Warsaw, IN, USA). Patients underwent routine post-operative clinical and radiographic evaluation (anteroposterior, lateral scapula, and axillary views) at 6 weeks, 3 months, 6 months, 1 year, and 2 years. Additional follow-up was provided when clinically indicated. Demographic data, including age, sex, comorbidities, and history of prior open or arthroscopic acromioplasty, were collected. In addition, the interval between acromioplasty and rTSA was recorded. Surgical indication was also collected. CTA was defined according to Neer et al^20^ as humeral head collapse with massive rotator cuff tears with or without osteoarthritis. Radiographic parameters were measured in a blinded manner on standardized anteroposterior radiographs obtained preoperatively and at six weeks post-operatively. Measurements were performed in accordance with the methods described in the original publications (J.V. and P.V.). Radiographic quality was assessed for all shoulder radiographs prior to analysis. Images were evaluated for adequacy of positioning, exposure, field of view, and visualization of relevant anatomical landmarks (including the glenoid, humeral head, acromion, and clavicle). Radiographs were considered acceptable if they met predefined criteria for standard anteroposterior projections (Grashay view). Imaging parameters (tube voltage, exposure time, and patient positioning) were standardized across all examinations. Bony quality was evaluated using AT^31^ and the DTI.^28^ Acromiohumeral distance (ACHD) represented distalization, and lateral acromion-to-greater tubercle distance (LAGT) represented lateralization. In addition, the CSA,^18^ LSA, and distalization shoulder angle (DSA)^5^ were quantified (Figure 1). Statistical analyses were performed using Statistical Package for the Social Sciences (v.27.0; IBM Corp., Armonk, NY, USA). Data normality was assessed with the Kolmogorov-Smirnov test and visual inspection of histograms. Continuous variables were analyzed with Student *t*-test and Mann-Whitney *U* test were applied to normally and non-normally distributed continuous variables, respectively. Categorical variables were compared using Fisher exact test. Multivariate Firth logistic regression was applied to assess predictors of ASF. A *P* value <.05 was considered statistically significant.

## Results

During the 6-year study period, 698 primary rTSAs were performed with a minimum follow-up of two years. Thirty-six stress fractures were identified corresponding to an incidence of 5.2%. Because of proximal humeral fractures and unavailable imaging, parameters including LSA, ACHD, and LAGT could only be collected for 621 shoulders.

### Demographic data

Among the 698 patients, 515 (73.8%) were females and 183 (26.2%) males. Six of the 36 patients with ASF were males (16.7%), with no significant difference in sex distribution (*P* = .181). Patient age ranged from 36 to 95 years (mean: 72.9 ± 8.6 years). Patients with ASF were slightly older (mean 74.2 ± 7.6 years) than those without (72.9 ± 8.6 years), though not significantly so (*P* = .278).

### Acromial stress fractures

The mean interval between rTSA and ASF onset was 164 days. Type I fractures were most common (52.8%), followed by Type II (41.6%) and Type III (5.6%).

### Comorbidities

Thirteen patients had rheumatoid arthritis, 4 had spondyloarthropathy, and 1 had psoriatic arthritis. None of these patients developed ASF.

#### Acromioplasty

A total of 111 patients (15, 9%) had undergone prior acromioplasty. All but 1 patient underwent arthroscopically acromioplasty. Among patients with ASF, 30.6% had a history of subacromial decompression, compared with 15% of those without ASF (*P* = .014). The mean interval between acromioplasty and rTSA was significantly longer in the ASF group (10.5 ± 6.63 years) than in the non-ASF group (6.22 ± 4.98 years, *P* = .026).

### Radiological parameters

#### Acromial thickness

Mean AT was significantly lower in the ASF group (6.5 ± 1.6 mm) than in the non-ASF group (7.7 ± 1.6 mm, *P* < .001) (Table I). No significant difference was observed between acromioplasty (7.3 ± 1.7) and nonacromioplasty group (7.69 ± 1.6).

**Table I:** Radiographic parameters.

Parameter	ASF group	Control group	*P* value
Acromial thickness (AT)	6.5 ± 1.6 mm	7.7 ± 1.6 mm	<.001
Deltoid tuberosity index (DTI)	1.29 ± 0.09	1.38 ± 0.13	<.001
Preoperative critical shoulder angle (CSA)	32.4 ± 4.3°	33.6 ± 4.9°	.23
Post-operative critical shoulder angle (CSA)	37.0 ± 5.6°	34.7 ± 6.5	.033
ΔCritical shoulder angle (CSA)	4.7 ± 6.9°	1.0 ± 4.9°	.001
Preoperative lateralization shoulder angle (LSA)	103.3 ± 9.4°	99.9 ± 9.8°	.068
Post-operative lateralization shoulder angle (LSA)	66.4 ± 35.7°	83.5 ± 9.8°	.034
Δ Lateralization shoulder angle (LSA)	17.6 ± 14.4°	16.4 ± 12.6°	.58
Preoparative distalization shoulder angle	26.8 ± 8.5°	27.9 ± 7.9°	.255
Post-operative distalization shoulder angle	46.5 ± 10.9°	45.8 ± 8.9	.584
Δ Lateralization shoulder angle	19.7 ± 10.4°	18.0 ± 9.3°	.184
Acromiohumeral distance (ACHD)	6.6 ± 4.2 mm	6.9 ± 2.9 mm	.13
Lateral acromion-to-greater tubercle distance (LAGT)	31.5 ± 7.4	32.3 ± 8.2 mm	.298

*ASF*, acromial scapular stress fracture.

#### Deltoid tuberosity index

Mean DTI was significantly lower in patients with ASF (1.29 ± 0.09) compared with those without ASF (1.38 ± 0.13, *P* < .001) (Table I).

#### Critical shoulder angle

The mean preoperative CSA was 32.4 ± 4.3° in the ASF group and 33.6 ± 4.9° in the non-ASF group (*P* = .23). Post-operative CSA was significantly higher in the ASF group (37.0 ± 5.6°) compared with the non-ASF group (34.7 ± 6.5°; *P* = .033). The mean change in critical shoulder angle (ΔCSA) was also greater in the ASF group (4.7 ± 6.9° vs. 1.0 ± 4.9°; *P* = .001) (Table I).

#### Lateralization shoulder angle

Preoperatively, the mean LSA was 103.3 ± 9.4° in the ASF group and 99.9 ± 9.8° in the non-ASF group (*P* = .068). Post-operatively, LSA was significantly lower in the ASF group (66.4 ± 35.7°) compared with the non-ASF group (83.5 ± 9.8°; *P* = .034). The mean ΔLSA did not differ significantly between groups (*P* = .58) (Table I).

### Distalization shoulder angle

Pre-operative and post-operative DSA values neither differ significantly between groups (preoperative *P* = .255; post-operative *P* = .584) nor did the mean change in DSA (*P* = .184) (Table I).

#### Acromiohumeral distance

No significant differences were found pre-operatively and post-operatively or for ΔACHD (Table I).

#### Lateral acromion-to-greater tuberosity distance

Both pre-operative and post-operative measurements were similar between groups, with no significant differences in ΔLAGT (Table I).

### Surgical indication

In the non-ASF group, 563 patients (85%) had CTA as the primary indication for surgery, compared 32 patients (88.9%) in the ASF group (*P* = .527). Other indications, including fracture, osteoarthritis, and avascular necrosis, showed no significant differences between groups.

### Firth logistic regression analysis of risk factors for stress fracture

Firth logistic regression was used to reduce small-sample bias and to provide reliable estimates in the presence of rare events, such as ASF, which can cause separation in standard logistic regression models. Firth logistic regression was used to assess the association between clinical and radiographic factors and the risk of post-operative stress fracture. Acromioplasty was associated with a higher odds of stress fracture (odds ratio [OR] 2.16, 95% confidence interval [CI] 0.94–4.70), although this did not reach statistical significance (*P* = .070), indicating a trend toward increased risk. Female sex was not significantly associated with stress fracture (OR 1.17, 95% CI 0.42–2.96, *P* = .751). Higher DTI values were significantly protective against stress fracture (OR 0.61, 95% CI 0.45–0.80, *P* < .001). AT was not significantly associated (OR 1.15, 95% CI 0.96–1.49, *P* = .487). Finally, ΔCSA was significantly associated with increased odds of stress fracture (OR 1.14, 95% CI 1.06–1.22, *P* < .001).

Firth's penalized logistic regression was used to assess predictors of stress fractures. In sensitivity analyses excluding AT, ΔDCSA, or sex, acromioplasty consistently increased the risk of stress fracture, with ORs ranging from 2.12 to 2.18 and borderline significance (*P* ≈ .058–.067). DTI remained protective in all models, with ORs of 0.49–0.53 (*P* < .001). Excluding ΔDCSA, AT was not significantly associated with stress fracture (OR 1.11, *P* = .488). Excluding AT, DCSA remained a significant risk factor (OR 1.14, *P* < .001). Excluding sex, the results for acromioplasty, DTI, and the remaining variables were essentially unchanged, and female sex was not a significant predictor in models that included it. These sensitivity analyses demonstrate that the associations of acromioplasty, DTI, and DCSA with stress fracture risk are robust to exclusion of individual predictors (see Supplementary Data).

## Discussion

rTSA is an established procedure with expanding indications, and its global use continues to increase.^2^^,^^9^^,^^32^ However, ASF remain a major complication, leading to persistent functional impairment and poor clinical outcomes.^4^^,^^12^^,^^21^^,^^24^ In this cohort, ASF occurred in 5.2% of patients, consistent with previously reported incidences ranging from 2.9% to 10%.^11^^,^^14^^,^^16^

Although female sex has been proposed as a risk factor,^6^^,^^15^^,^^16^^,^^25^ the higher proportion of women in our ASF group did not reach statistical significance. Similarly, patient age showed no significant association, supporting the hypothesis that age may act as a surrogate for bone density rather than an independent risk factor.^6^

Rheumatoid arthritis has been reported to increase ASF risk,^6^^,^^17^^,^^25^^,^^26^ yet none of the patients with RA in our series developed in ASF. This may reflect earlier diagnosis and aggressive disease control in Belgium, where RA disease activity tends to be lower compared with international cohorts.^7^

The influence of prior acromioplasty on ASF development remains controversial.^6^ A recent large-scale registry study involving 106,599 patients found that subacromial decompression increased ASF risk by 26%, though laterality between acromioplasty, rTSA, and ASF was assumed rather than confirmed.^27^ In our cohort, patients with a history of acromioplasty exhibited a trend toward reduced AT compared with those without prior surgery, although this difference did not reach statistical significance. Similarly, Firth logistic regression analysis demonstrated that prior acromioplasty was associated with increased odds of ASF, yet the association did not achieve conventional levels of statistical significance. Notably, the interval between acromioplasty and rTSA was significantly longer in patients who subsequently developed ASF. This observation may reflect a cumulative effect of progressive acromial thinning over time, potentially exacerbated by underlying CTA, thereby increasing the mechanical vulnerability of the acromion to stress fractures following rTSA. Taken together, these findings underscore the importance of considering both the timing of prior acromial surgery and ongoing degenerative changes when evaluating individual fracture risk.

Radiographic predictors of ASF remain inconsistently defined. Both AT and DTI, indicators of local bone quality^28^^,^^31^ were significantly lower in patients with ASF. Previous studies have shown a similar trend,^12^^,^^13^ although findings regarding DTI have been inconsistent, possibly due to inclusion of traumatic fractures or stress fractures in earlier cohorts.^12^^,^^13^

CSA is a valuable radiographic measure in shoulder pathology.^22^ Post-operative CSA and ΔCSA were significantly higher in ASF patients, supporting the hypothesis that increased CSA or excessive alteration of CSA during surgery may elevate fracture risk. As suggested by Kriechling et al,^12^^,^^13^ baseplate positioning influences CSA, with inferior tilt and lateralization reducing CSA. Surgeons should therefore avoid excessive modification of CSA intraoperatively.

Similarly to some prior reports,^12^^,^^13^ there were no differences observed for post-operative LSA, ACHD, or LAGT. These findings emphasize that ASF pathogenesis is multifactorial, involving both mechanical and biological components.

Although CTA has been cited as a contributing factor,^11^^,^^25^ its prevalence did not differ significantly between groups in this study.

Multivariate analysis confirmed DTI and ΔDCSA as independent predictors of ASF. These findings underscore the importance of comprehensive pre-operative and post-operative evaluation to identify high-risk patients and tailor surgical planning accordingly.

Study limitations include lack of data on smoking status and body mass index, both potential contributors to bone quality.^6^ The exact role of body mass index and smoking on the development of ASF is not yet established. This study is limited to its retrospective design.

## Conclusion

Preoperative DTI and change in CSA were significantly associated with the development of ASFs following primary rTSA. Prior acromioplasty does not significantly increases the odds of ASF.

Collectively, these data highlight the need for individualized surgical planning. Careful assessment of DTI and ΔCSA may help identify patients at elevated risk, reduce the incidence of ASF, and improve long-term outcomes after rTSA.

## Disclaimers:

Funding: No funding was disclosed by the authors.

Conflicts of interest: The author, their immediate family, and any research foundation with which they are affiliated have not received any financial payments or other benefits from any commercial entity related to the subject of this article.

Patient consent: Obtained.

## Supplementary Data

Article Processing Charge

Sensitivity Analysis
