# Supplementary material for: Risk factors for acromial stress fractures following primary reverse total shoulder arthroplasty: the impact of prior acromioplasty and radiographic parameters
Source: J Shoulder Elb Arthroplast. 2026 May 12;10(1-2):100016. doi: 10.1016/j.jsea.2026.100016 (PMC13196051; doi:10.1016/j.jsea.2026.100016)
Supplement: Sensitivity Analysis [file mmc2.docx]

Sensitivity analysis

| **Predictor** | **OR (risk)** | **Lower 95% CI** | **Upper 95% CI** | **p-value** | **Model** |
| --- | --- | --- | --- | --- | --- |
| **Intercept** | 0.94 | 0.15 | 6.69 | 0.951 | Full |
| **Acromioplasty** | 2.20 | 0.99 | 4.57 | 0.058 | Full |
| **Geslacht** | 1.17 | 0.41 | 2.96 | 0.728 | Full |
| **DTI** | 0.61 | 0.46 | 0.79 | <0.001 | Full |
| **AT** | 1.15 | 0.67 | 1.49 | 0.488 | Full |
| **DCSA** | 1.14 | 1.06 | 1.22 | <0.001 | Full |
| **Acromioplasty** | 2.18 | 1.06 | 4.76 | 0.066 | Without AT |
| **Geslacht** | 1.19 | 0.41 | 2.99 | 0.729 | Without AT |
| **DTI** | 0.61 | 0.45 | 0.80 | <0.001 | Without AT |
| **DCSA** | 1.14 | 1.06 | 1.22 | <0.001 | Without AT |
| **Acromioplasty** | 2.12 | 0.99 | 4.56 | 0.067 | Without DCSA |
| **Geslacht** | 1.05 | 0.41 | 2.73 | 0.924 | Without DCSA |
| **DTI** | 0.60 | 0.45 | 0.77 | <0.001 | Without DCSA |
| **AT** | 1.11 | 0.70 | 1.44 | 0.615 | Without DCSA |
