# Supplementary material for: Risk factors for acromial stress fractures following primary reverse total shoulder arthroplasty: the impact of prior acromioplasty and radiographic parameters
Source: J Shoulder Elb Arthroplast. 2026 May 12;10(1-2):100016. doi: 10.1016/j.jsea.2026.100016 (PMC13196051; doi:10.1016/j.jsea.2026.100016)
Supplement: Article Processing Charge [file mmc1.docx]

Dear Editor-in-Chief,

I hope this message finds you well.

I am writing to respectfully request a waiver of the Article Processing Charge (APC) for my manuscript entitled “Risk Factors for Acromial Stress Fractures Following Primary Reverse Total Shoulder Arthroplasty: The Impact of Prior Acromioplasty and Radiographic Parameters” which has been submitted to your esteemed journal for consideration.

This study represents my original research work conducted during my residency training. As a medical resident, I undertook this project independently without any external funding, grants, or institutional financial support. The research was completed with the aim of contributing meaningful findings to the scientific community and advancing knowledge in our field.

Unfortunately, due to the absence of funding and my current position as a resident with limited financial resources, I am unable to cover the APC from my personal income. Paying the required fee would pose a significant financial burden.

I respectfully request your kind consideration for a full or partial waiver of the APC. I am fully committed to complying with all other journal requirements and revisions, and I sincerely hope that the financial constraints will not hinder the dissemination of this work.

Thank you very much for your time and consideration. I greatly appreciate the opportunity to submit my work to your journal and look forward to your response.

Yours sincerely,
